# Effect of dissolved-gas concentration on bulk nanobubbles generation using ultrasonication

**DOI:** 10.1038/s41598-020-75818-8

**Published:** 2020-11-02

**Authors:** Jeong Il Lee, Byung-Seung Yim, Jong-Min Kim

**Affiliations:** 1 School of Mechanical Engineering, Chung-Ang University, Seoul, 156-756 Korea; 2 School of Mechanical System Engineering, Kangwon National University, Gangwon-do, 25913 Korea

**Keywords:** Nanoparticles, Synthesis and processing

## Abstract

In this study, the effects of dissolved-gas concentration in liquid water on the nucleation and growth of bubbles and nanobubble (NB) generation were investigated by measuring the concentration and size distribution of NBs. Three types of liquids with different dissolved-gas concentrations—undersaturated, saturated, and supersaturated deionized (DI) water—were prepared, and NBs were generated via ultrasonic irradiation. As the dissolved-gas concentration increased, a large number of bubbles with relatively large diameters (several tens of micrometers or more) were generated, but the NB concentration decreased. The surface tension decreased with an increase in the dissolved gas concentration, and thus, the tensile strength which required for bubble growth became lower. Therefore, there were barely any NBs in supersaturated conditions because of the accelerated nucleation and bubble growth.

## Introduction

In recent years, nanobubbles (NBs) have attracted considerable attention because of their potentials^1^. They exist in two forms within a liquid: surface nanobubbles (SNBs), which are typically trapped on non-flat surfaces, and bulk nanobubbles (BNBs), which are dispersed and suspended in solutions. NBs are gaseous bubbles that are typically tens to hundreds of nanometers in size. These tiny NBs remain in the solution for a long time owing to their extremely low (negligible) floating rates, and thus, NB solutions have a variety of physicochemical properties^2^. Because of the extraordinary properties of NB solutions, NBs have been used in various fields such as growth promotion of plants^3^, ultrasonic reflection^4^, drug delivery^5^, high efficiency fuel^6^, and cleaning^7^. There are many questions concerning the existence and stability of NBs. The internal pressure of bubble with 100 nm in radius is about 15 atm from the Young–Laplace equation^8^. Therefore, according to Epstein-Plasset theory the bubble should be rapidly dissolved and disappeared into the liquid within 80 µs^9,10^. On the contrary, Hemmingsen et al.^11^ reported that it is not appropriate to estimate the internal pressure of NB through the Young–Laplace equation because surface tension is greatly affected by the interface curvature and the internal gas pressure of NB. In addition, Tolman et al.^12^, theoretically calculated the surface tension of the droplets. They reported that surface tension significantly decreases as the bubble size decreases, and thus the internal pressure of NB will be also lower than the calculated value. In addition, there are a number of studies that provide the existence of BNBs through an experimental approach. Ohgaki et al.^13^ produced the NB replica through rapid cryogenic freezing and fracture of NB solution. The NB replica was imaged by scanning electron microscopy (SEM). Uchida et al.^14^ also produced NB replica in a similar way and it was imaged by transmission electron microscopy (TEM).

NBs can be produced by various methods such as capillary, gas–liquid mixing, and cavitation^15^. Among the NB generation methods using cavitation, the acoustic cavitation method is based on a phenomenon that generates bubbles through exposure to periodically repeated sound waves with positive and negative pressure; nucleation and expansion of bubbles occur in a solution under the negative-pressure condition, whereas the grown bubbles are contracted under the positive-pressure condition. The bubbles generated with ultrasound slowly grow under periodic expansion and contraction environments, and finally implode when they attain a critical size^16^. Among the fragmented bubbles, some diffuse into the liquid and disappear, whereas others become NBs through refinement and stabilization behaviors. Yasuda et al.^17^ reported the effects of ultrasonic frequency and power on the generation of NBs. In their study, the BNBs were generated by ultrasonication to ultrapure water, and the concentration of NBs increased under the low-frequency and high-power conditions of ultrasound.

Bubble generation via acoustic cavitation was discovered in the mid-nineteenth century and has been studied for a long time by several researchers. However, research on bubble generation using acoustic cavitation has been dominantly focused on micro/macro-sized bubbles, which are visible to the naked eye; furthermore, limited research has been conducted on the generation and growth of nano-sized bubbles using ultrasound. Moreover, no experimental report on the effect of dissolved-gas concentration on NB generation using ultrasonication is available.

This study was focused on determining the influence of dissolved-gas concentration on NB generation using ultrasonication through NTA. An image of the bubble growth state was captured, and the surface tension were measured at different gas concentrations. The results indicate that the NB concentrations decreased as the dissolved-gas concentration increased. In this paper, the nucleation and growth of the bubbles under different gas concentrations are discussed.

## Methods

Three types of liquids with different dissolved-gas concentrations—undersaturated, saturated, and supersaturated DI water—were prepared to evaluate the effects of dissolved-gas concentration in liquids on the NB-generation behavior. The DI water was produced using a water-purification system (Pure Power III+, Hunan, Korea), which has an electrical conductivity of 0.32 ± 0.14 µs/cm and pH of 5.93 ± 0.01. The oxygen gas (purity: 99.999%, Shinyoung Gas Co., Korea) was used to control the dissolved-gas concentration.

To prepare the undersaturated liquid, the DI water was degassed through heating using an oil bath for approximately three days at a constant temperature of 60 °C and atmospheric pressure. The saturated liquid used DI water at room temperature and atmospheric pressure. The supersaturated liquid was prepared by supplying oxygen gas to DI water using a pressurized tank at 2 atm for 24 h. The concentration of dissolved oxygen (DO) in each of the prepared liquids was measured using a DO meter (Thermo Scientific Orion Versa Star, Thermo Scientific, USA). Also, the zeta potential of each test liquid were measured using a zeta potential analyzer (ZetaPALS, Brookhaven Instruments, USA) to examine the electrokinetic properties of the generated particle (contamination or bubble) during the each test liquid preparation.

To generate NBs within the liquid, 500 ml of each type of the prepared liquid was placed in a beaker, and ultrasound was irradiated for 10 min at 20 kHz and 1100 W using a piezo-type ultrasonic horn booster (Daehan Ultrasonic Engineering., Korea). While ultrasound was irradiated, the temperature was maintained at room temperature using a cooling bath to prevent an increase in the temperature of the liquid. After the ultrasonic irradiation, the samples were sealed in glass vials to prevent the permeation of external contaminants, and then, the samples were left for 30 min to remove bubbles over micro size.

The size distribution and concentration of the ultrasound-generated NBs were measured using NTA system (NanoSight LM10-HSBFT14, Malvern, UK) with a blue polarized 405 nm laser. The NB concentration is the total number of NBs, 10–1000 nm in diameter, per milliliter. Also, zeta potential measurement were performed using zeta potential analyzer to investigate the existence and short-term stability of NBs. Each measurement was recorded five times.

## Results and discussion

**Figure 1:**
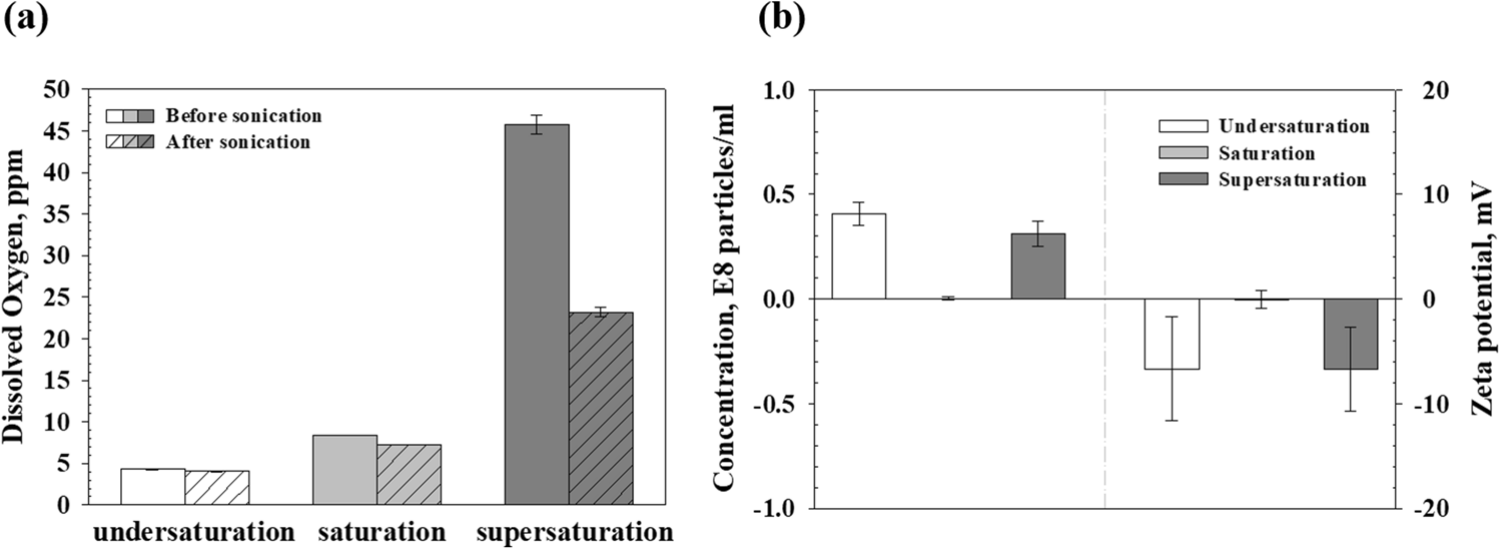
Results of each measurement of DI water with respect to the dissolved-gas concentration; (**a**) Dissolved oxygen (DO) concentration before and after sonication, and (**b**) nanobubble (NB) concentration (left) and zeta potential (right) before sonication.

Figure 1a presents the DO concentrations of liquids with different dissolved gas concentrations before and after sonication. As presented in the results, the undersaturated DI water exhibited a relatively low DO concentration of 4.29 ± 0.03 ppm caused by the effective degassing. The DO concentration of saturated DI water was 8.33 ± 0.02 ppm. The supersaturated DI water exhibited a relatively high DO concentration of 45.74 ± 1.18 ppm because of the excessive oxygen gas under the pressurized environment. After sonication, the DO concentration (undersaturated DI water: approximately 4.03 ± 0.03 ppm, saturated DI water: approximately 7.23 ± 0.02 ppm, and supersaturated DI water: approximately 23.21 ± 0.60 ppm) in all liquids decreased because the irradiating ultrasound in liquid is the one of the degassing methods^18^. The difference of DO concentration in each undersaturated and saturated DI water before and after sonication was insignificant. Although there is a large difference in DO concentration of approximately 22 ppm after sonication to the supersaturated DI water, it still remains oversaturated condition. Figure 1b presents the number of initially existing bubbles and their zeta potential in each of the prepared liquids. The NTA results of each type of DI water indicate that the initial particle concentration in the undersaturated DI water before ultrasonic irradiation was 0.41 ± 0.02 × 10^8^ particles/ml and mean diameter was 250 nm. Also, the undersaturated DI water exhibited zeta potential of − 6.66 ± 4.95 mV. It is well known that bubble present in water (no addition of electrolyte or surfactant) have electrokinetic property which usually exhibit negative charge. Although it is smaller than the zeta potential value of the stabilized bubble^19,20^, it has a negative charge. Therefore, it is expected that the bubbles were generated during the sample preparation, but they existed in an unstable condition. These preexisting NBs attributed to the bubble growth instigated by the diffusion of dissolved gas into the nucleus, which was caused by boiling nucleation during liquid heating^21^ or the trapped and detached bubbles on vessel surfaces when the liquid was poured^22^. In contrast, the bubbles concentrations of the saturated DI water were barely observed. The initial bubbles concentration within the supersaturated DI water was 0.31 ± 0.06 × 10^8^ particles/ml and mean diameter was 316 nm. The zeta potential was − 6.69 ± 3.96 mV. It is also considered that the bubbles is unstable state similar to those in undersaturated DI water. These bubbles were caused by the heterogeneous nucleation that occurred in a non-flat surface of the vessel when the liquid under the pressurized environment was exposed to atmospheric pressure^23^.

**Figure 2:**
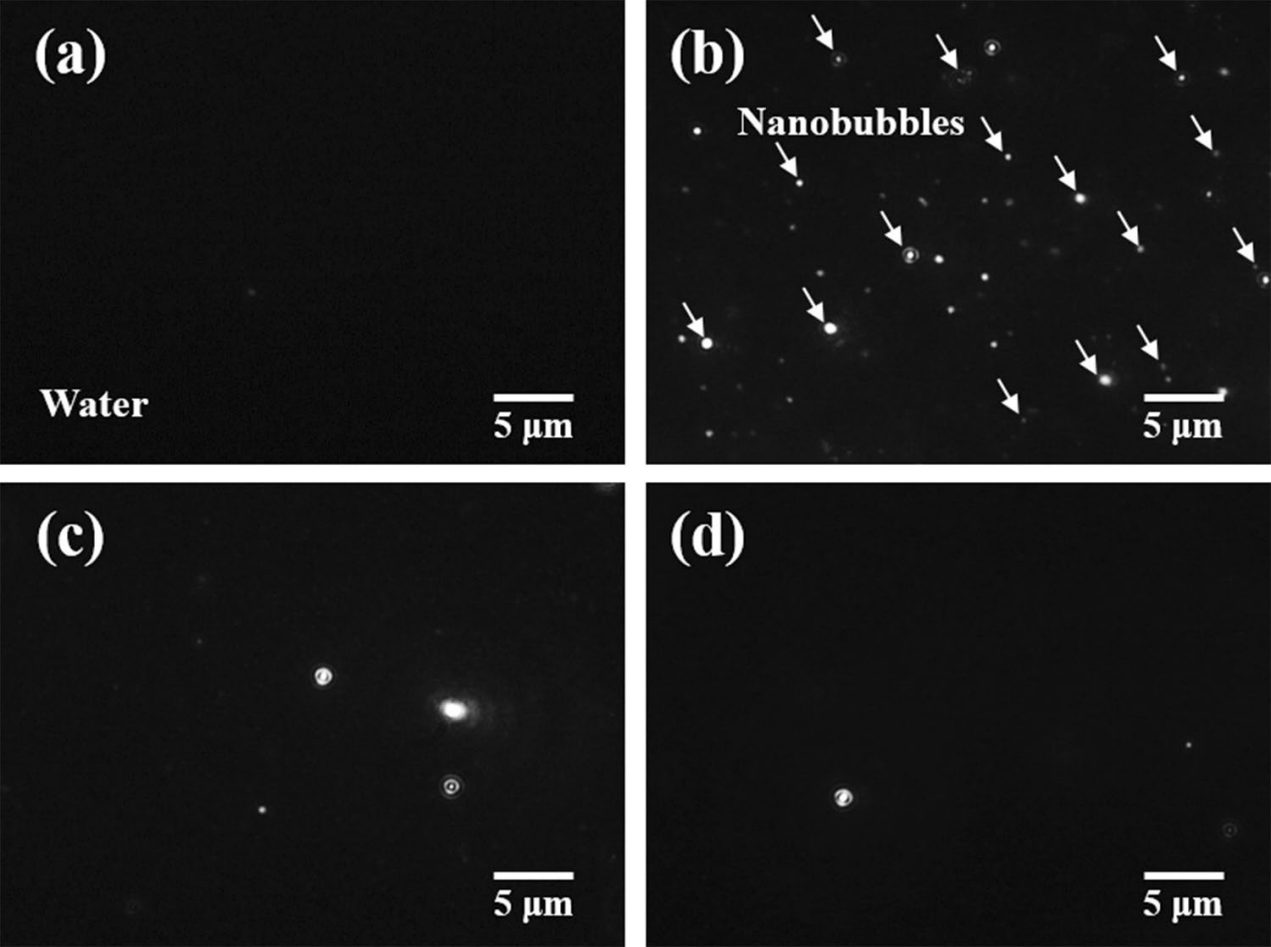
(**a**) NBs in saturated DI water before sonication; NBs in (**b**) undersaturated, (**c**) saturated, and (**d**) supersaturated DI water after sonication.

Figure 2 presents the shapes of the NBs in each type of DI water before and after sonication. In the image, the black background represents the DI water, and the white dots represent the NBs. The NBs were not observed in the saturated DI water without ultrasonication (Fig. 2a), but they existed in all the three types of DI water after ultrasonication (Fig. 2b–d). This phenomenon indicates that NBs were generated through the growth of nucleated bubbles and the stabilization process after the occurrence of acoustic cavitation caused by sound waves^19^. In particular, as illustrated in Fig. 2b, when ultrasound was irradiated onto the undersaturated DI water, a relatively large number of NBs was generated in comparison with those in saturated and supersaturated DI water.

**Figure 3:**
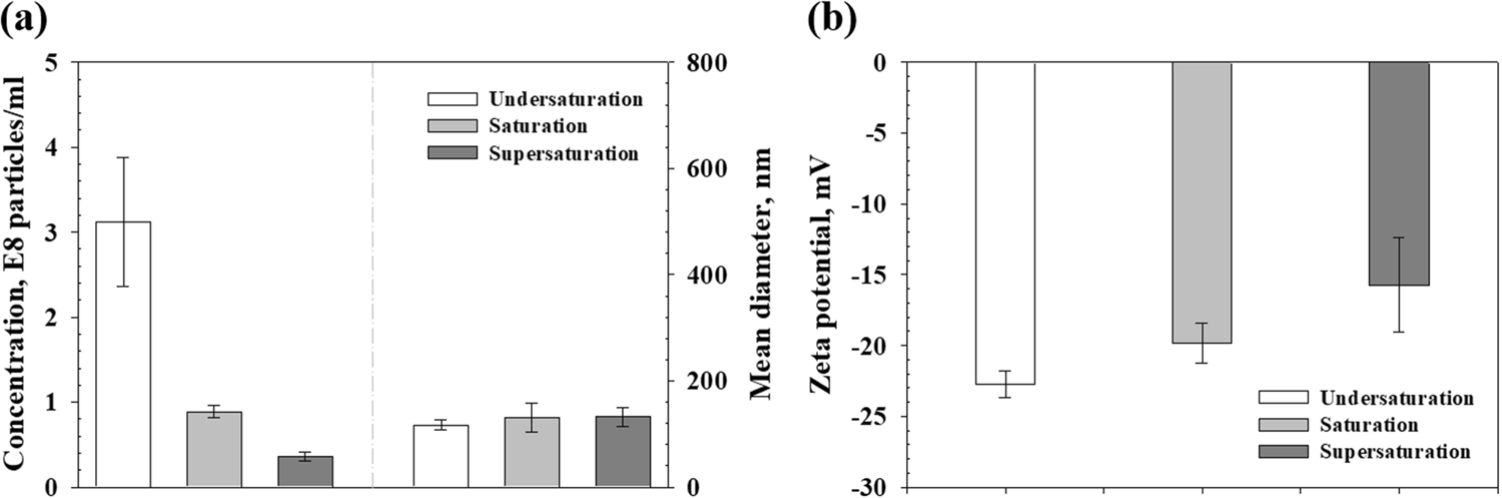
Results of each measurement of DI water with respect to the dissolved-gas concentrations after sonication; (**a**) NB concentration (left) and mean diameter (right), and (**b**) zeta potential.

Figure 3 shows the NTA and zeta potential analysis of the generated NBs in each type of DI water with different dissolved-gas concentrations after ultrasonic irradiation. Figure 3a shows the concentration and mean diameter of the generated NBs. For the undersaturated DI water, the total NB concentration was 3.12 ± 0.76 × 10^8^ particles/ml, which implies that a large number of NBs were generated (approximately 2.71 × 10^8^ particles/ml) compared to the preexisting NBs in the undersaturated DI water before the ultrasonic irradiation. For the saturated DI water, the total NB concentration was 0.89 ± 0.07 × 10^8^ particles/ml, and a relatively small number of NBs were generated (approximately 0.69 × 10^8^ particles/ml). On the other hand, the total NB concentration in the supersaturated DI water was 0.36 ± 0.06 × 10^8^ particles/ml, and the NBs were not nearly generated. Meanwhile, the mean diameters of NB for the three types of DI water were almost the same (undersaturated DI water: approximately 116.67 ± 9.60 nm, saturated DI water: approximately 139.66 ± 3.78 nm, and supersaturated DI water: approximately 132.66 ± 17.61 nm). Figure 3b shows the zeta potential of each liquid after sonication. As mentioned above, the negative zeta potential is the one of the main factors supporting the existence and stability of NBs in liquid^22,24^. The results indicate that all samples have negative charged potential (undersaturated DI water: approximately − 22.72 ± 0.91 mV, saturated DI water: approximately − 19.83 ± 1.39 mV, and supersaturated DI water: approximately − 15.71 ± 3.36 mV). Although there is little difference in the zeta potential, it shows a value of − 15 mV or less in all cases. Through these result, it is considered that the NBs generated by ultrasonication exist in a relatively stable state.

**Figure 4:**
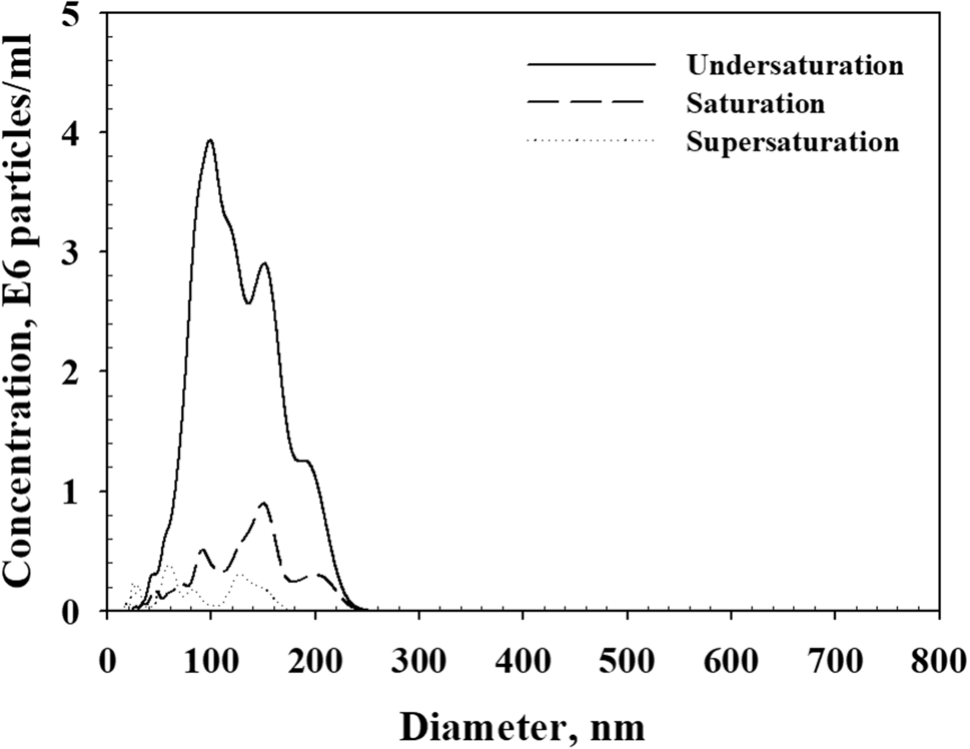
Size distribution of the generated NBs in DI water with different dissolved-gas concentrations after sonication.

Figure 4 presents the size distribution of the generated NBs within each type of DI water. As presented in the results, the sizes of NBs in each type of DI water were in the range of 0–250 nm, and showed multiple peaks in all the cases.

From the considerable research conducted on the cavitation bubble, it is well known that a large number of bubbles can be easily generated by increasing the gas solubility^25–27^. Thus, it can be thought that the NBs can be generated more effectively through ultrasound irradiation onto the solution with a high dissolved-gas concentration. However, the generated NB concentration increased as the dissolved-gas concentration decreased.

The result indicate that the bubble growth accelerated, and the NB size exceeded the micro size as the dissolved-gas concentration was increased in ultrasonic irradiation environment. In general, most liquids contain dissolved gases because most, if not all, of these dissolved gases cannot be possibly eliminated from the liquid. Accordingly, Brennen^15^ and Apfel^28^ reported that the Young–Laplace equation with respect to the internal pressure of bubbles (related to nucleation and bubble growth) should involve the sum of partial pressures of the dissolved gas. For bubble growth, nucleation must occur, and an appropriate tensile strength must be applied. The tensile strength required for bubble growth can be represented by Eq. (1)^15,28^.

1 $$P_{V} - P = 2S/R - P_{G}$$

where *P*_*V*_, *P*, *S*, *R* and *P*_*G*_ are the vapor pressure, liquid pressure, surface tension, nucleated bubble radius, and sum of partial pressures of the gas in the nucleated bubbles, respectively. Moreover, *P*_*V*_ − *P* (i.e., *ΔP*) is the tensile strength required for bubble generation and growth. It can be inferred from the relationship between the parameters in Eq. (1) that the tensile strength (*P*_*V*_ − *P*) required for bubble growth is influenced by the surface tension (*S*) and sum of partial pressures of gas in the nucleated bubbles (*P*_*G*_). In addition, Rooze et al.^25^ reported that the tensile strength is affected by the surface tension due to the dissolved-gas concentration.

Accordingly, a surface tension test for DI water with different dissolved-gas concentrations and bubble growth observation during ultrasonication were conducted to identify the causes of the decrease in NBs concentrations in the liquid with an increase in the dissolved-gas concentration.

To measure the surface tension of DI water according to the dissolved-gas concentration, a surface-tension analyzer (DTS 60, Surface Electro Optics, Korea) with a Wilhelmy plate was used. Each prepared liquid sample (300 ml) was placed in the beaker, and then, the cleaned thin platinum plate was immersed perpendicularly to the surface of the liquid to wet the surface of the plate. After wetting of the plate surface, the plate was pulled upwards, and the force acting on the plate by the wetted liquid was measured using a tensiometer. To obtain reliable results, the surface tension of each liquid was measured 10 times.

To investigate the difference in bubble growth during ultrasonic irradiation according to the dissolved-gas concentration, a digital single-lens reflex camera (ILCE-7M3, Sony, Japan) equipped with a telephoto zoom lens (FE 100–400 mm, Sony, Japan) was used. To obtain a visually identifiable image, light was projected from the bottom of the vial after entirely blocking the external light. Then, the bubble-growth behavior was observed through 10 continuous shooting modes per second.

**Figure 5:**
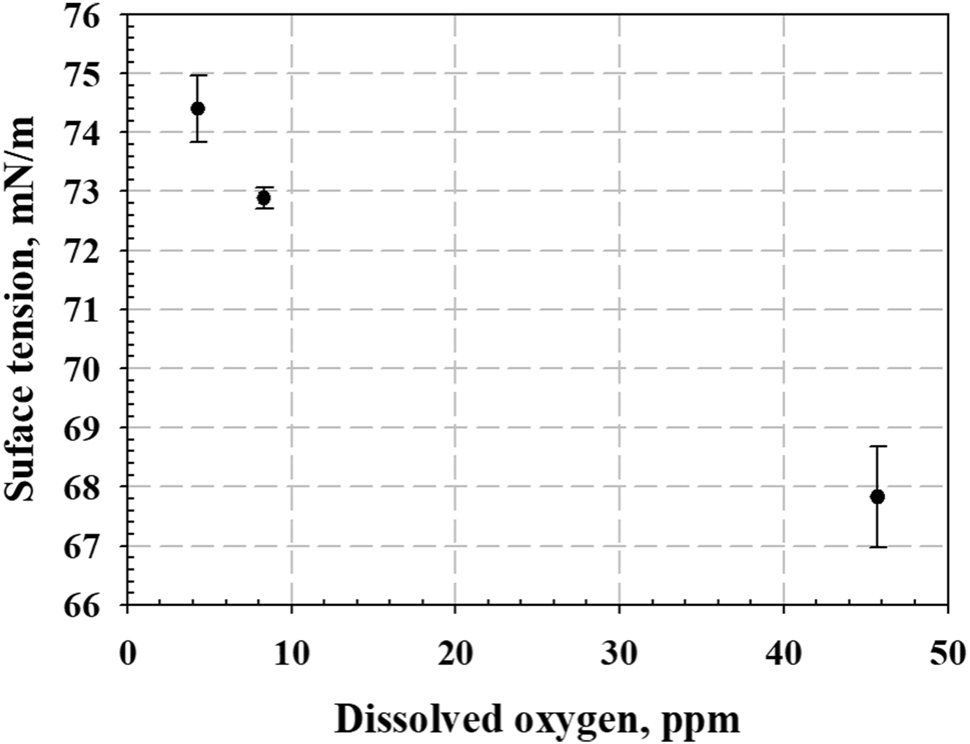
Surface tension of each type of DI water with different dissolved-gas concentrations.

The results of surface tension measurement for the DI water with different dissolved-gas concentrations are presented in Fig. 5. The results indicate that the surface tension of the DI water decreased with increase in the dissolved-gas concentration (undersaturated DI water: approximately 74.40 ± 0.02 mN/m, saturated DI water: approximately 72.89 ± 0.17 mN/m, and supersaturated DI water: approximately 67.82 ± 0.86 mN/m). The dissolved gas in the DI water acted like a surfactant, which reduced the force of attraction between the water molecules. According to Lubetkin^29^, the surface tensions of a liquid with dissolved gas and pure liquid are different, and the surface tension significantly decreases as the dissolved-gas concentration increases. This tendency appears stronger in highly soluble gases. Moreover, other researcher reported that the high dissolved gas concentration in liquids can increase the nucleation rates^29–31^ and decrease the cavitation threshold^32^. Therefore, multiple simultaneous nucleation easily occurred in the liquid with high dissolved-gas concentration. This can be attributed to the high nucleation rate due to a high dissolved gas concentration^29–31^; the surrounding dissolved gases gathered in the generated nuclei and increased the *P*_*G*_ value. Therefore, the increased *P*_*G*_ and decreased surface tension significantly reduced the tensile strength required for bubble growth, as shown in Eq. (1). When ultrasound was irradiated onto the supersaturated DI water, the tensile strength required for the nucleated bubble growth became lower. Consequently, the bubble growth accelerated with the increase in dissolved-gas concentration. And then the bubble growth accelerated again with rectified diffusion by unequal mass transfer and coalescence by Bjerknes force when the bubbles are exposed to repeated ultrasound waves in these multi-bubble system^33^. These multi-bubble can easily contact and combine each other (coalescence), because a large number of bubbles exist in supersaturated DI water^34,35^. Also individual bubbles rapidly grow due to the transfer of gases across the bubble interface (rectified diffusion), because a large amount of gas was still dissolved around the bubble interface in supersaturated DI water^36,37^. Therefore, the bubbles easily grew over the micro size when the ultrasound irradiated to high gas-dissolved liquid.

**Figure 6:**
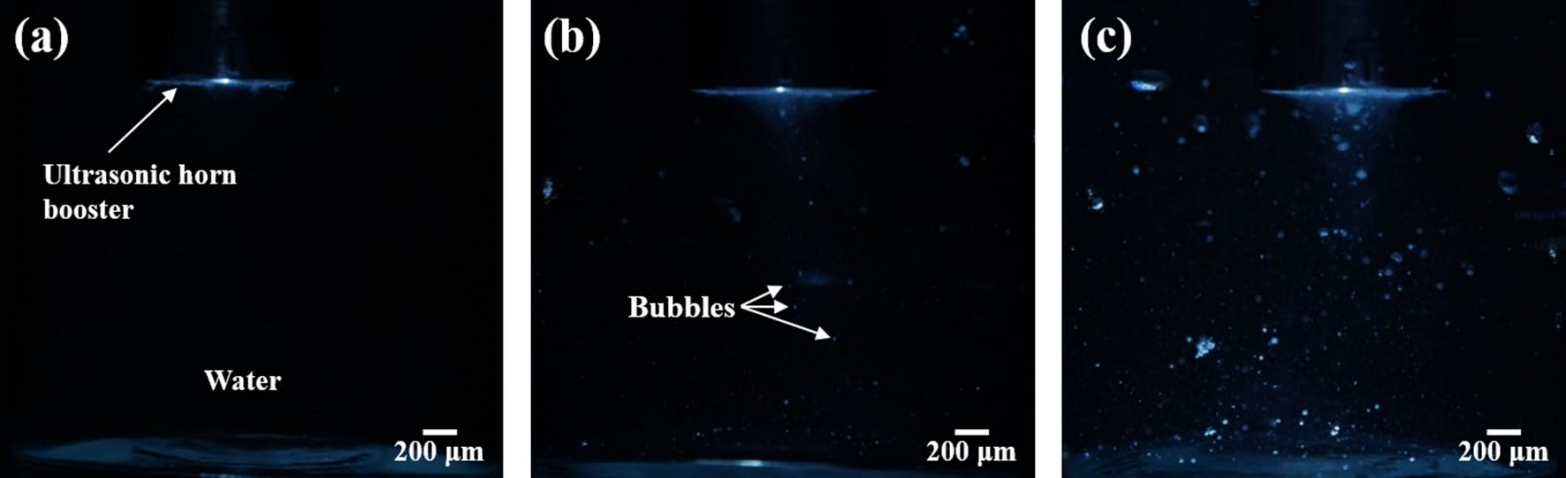
Bubble growth in (**a**) undersaturated, (**b**) saturated, and (**c**) supersaturated DI water during sonication.

This phenomenon is more clearly presented in the bubble-growth-observation results, as illustrated in Fig. 6. As shown in Fig. 6c, when the ultrasound was irradiated onto the supersaturated DI water, a large number of bubbles with relatively large diameters of several tens of micrometers or more were generated in the entire liquid. As mentioned earlier, this phenomenon was caused by the accelerated. According to Stokes’ equation, a 25 μm bubble rises upwards at a velocity of approximately 2.3 cm/min. Therefore, bubbles with a relatively large diameter of several tens of micrometers or more quickly rose to the surface of the liquid and then diffused in the atmosphere and disappeared. Thus, as indicated by the NTA results (Fig. 3), the NB concentrations of supersaturated DI water after ultrasonication were low because the sizes of most generated bubbles exceeded the micro size and thus quickly rose to the surface of the liquid. In contrast, as illustrated in Fig. 6a, in the undersaturated DI water, the bubbles were generated in a narrow region around the ultrasonic horn booster and the bottom of the liquid, and the size and concentration of the bubbles were very small in comparison with those of the supersaturated DI water (Fig. 6c). This phenomenon was a result of the suppressed bubble growth due to the low dissolved-gas concentration. As illustrated in Fig. 5, the undersaturated DI water has a relatively high surface tension owing to the low dissolved-gas concentration. Also, the undersaturated DI water has a relatively low nucleation rate in comparison with the supersaturated DI water^29–31^. Although the nucleation for NB generation occurred through ultrasonic irradiation and the surrounding dissolved-gas molecules began to accumulate in the generated nuclei, the undersaturated DI water had a low *P*_*G*_ value because the concentrations of the surrounding dissolved gases are relatively low in comparison with those in the case of supersaturated DI water. Therefore, as presented in Eq. (1), the tensile strength required for bubble growth increased owing to the high surface tension and low *P*_*G*_ value. Consequently, the nucleated bubble growth was suppressed because of the relatively high tensile strength after bubble nucleation. Although this condition is also considered to be a multi-bubble system, the number and size of bubble are expected to be much smaller than the bubbles of supersaturated DI water. Therefore, these bubbles are considered that it is hardly grow in an environment which is continuously irradiated ultrasonic waves. In other words, the bubble growth rate by coalescence and rectified diffusion extremely decreased compared with the bubbles in supersaturated DI water, because there are few bubble and low concentrated dissolved gas around the bubble interface in undersaturated DI water. Some of these bubbles diffused and disappeared in the water, and others existed as NBs through the stabilization process^38,39^. As a results, a large number of NBs can be easily generated and stably existed on undersaturated condition compared to supersaturated condition.

## Conclusions

In this study, the effect of dissolved-gas concentration on NB generation by ultrasonication was investigated. The NB concentration decreased with an increase in the dissolved-gas concentration. Further investigations into the surface tension of DI water with different dissolved-gas concentrations and bubble growth during ultrasonic irradiation indicate that the surface tension of DI water decreased as the dissolved-gas concentration increased. Because of the high dissolved gas concentration, the nucleation rate increased and surface tension decreased. As a result, the tensile strength required for bubble growth decreased. Therefore, when the supersaturated DI water was exposed to ultrasound irradiation, NBs were barely present in the DI water because bubble nucleation and growth were accelerated. Meanwhile, in the undersaturated DI water, NBs were generated in high concentrations because of the suppressed bubble growth due to the high surface tension and low *P*_*G*_ value. These results suggest that the dissolved-gas concentration in liquids is an important factor in the generation of NBs by ultrasonication.
